# Development of a Meshless Kernel-Based Scheme for Particle-Field Brownian Dynamics Simulations

**DOI:** 10.1021/acs.jpcb.4c01441

**Published:** 2024-07-10

**Authors:** Aristotelis P. Sgouros, Doros N. Theodorou

**Affiliations:** School of Chemical Engineering, National Technical University of Athens (NTUA), GR-15780 Athens, Greece

## Abstract

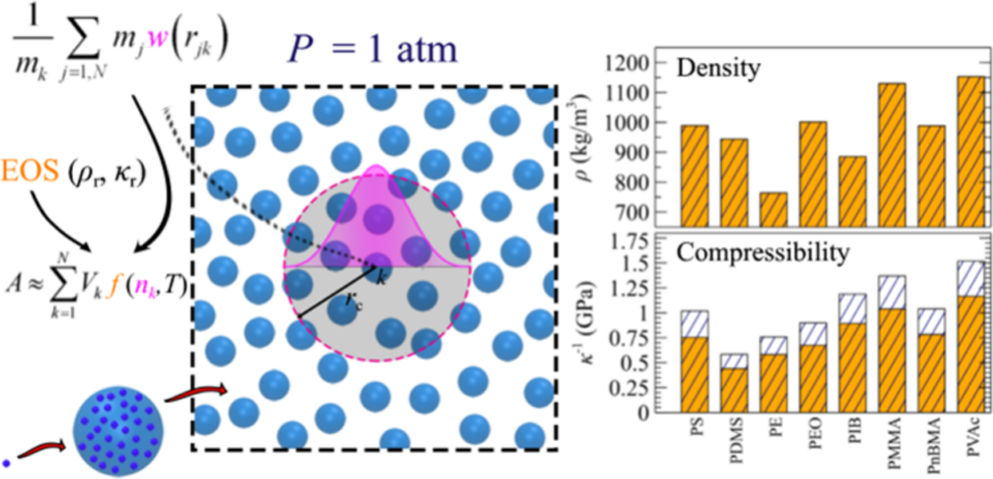

We develop a meshless discretization scheme for particle-field Brownian dynamics simulations. The density is assigned on the particle level using a weighting kernel with finite support. The system’s free energy density is derived from an equation of state (EoS) and includes a square gradient term. The numerical stability of the scheme is evaluated in terms of reproducing the thermodynamics (equilibrium density and compressibility) and dynamics (diffusion coefficient) of homogeneous samples. Using a reduced description to simplify our analysis, we find that numerical stability depends strictly on reduced reference compressibility, kernel range, time step in relation to the friction factor, and reduced external pressure, the latter being relevant under isobaric conditions. Appropriate parametrization yields precise thermodynamics, further improved through a simple renormalization protocol. The dynamics can be restored exactly through a trivial manipulation of the time step and friction coefficient. A semiempirical formula for the upper bound on the time step is derived, which takes into account variations in compressibility, friction factor, and kernel range. We test the scheme on realistic mesoscopic models of fluids, involving both simple (Helfand) and more sophisticated (Sanchez–Lacombe) equations of state.

## 1 Introduction

The advent of mesoscopic simulations has proven essential for tackling complex problems that involve intricate interactions and dynamics across multiple time and length scales.^1,2^ Mesoscopic simulations have facilitated immensely the optimization of industrial processes,^3^ the prediction of rheological properties of high molar mass polymers,^4,5^ and the study of phase separation,^3^ biomolecular systems,^6,7^ and fracture phenomena.^8,9^

A variety of mesoscopic simulation approaches have been developed, including particle-based models [Brownian dynamics (BD), coarse-grained molecular dynamics (CGMD),^3^ and dissipative particle dynamics (DPD)^10−16^], field-based models [density functional theory (DFT),^17,18^ self-consistent field theory (SCFT^19−21^), dynamic SCFT,^22^ and lattice Boltzmann methods^23,24^], and hybrid particle-field models [smooth particle hydrodynamics (SPH),^25^ smooth dissipative particle dynamics (SDPD),^26,27^ many-body dissipative particle dynamics (MDPD),^13−15,27^ hybrid molecular dynamics/self-consistent field schemes (MD-SCF),^28−32^ and hybrid Brownian dynamics/kinetic Monte Carlo (BD/kMC) schemes^7,33^ accounting for chain reptation^34−38^].

In particle-field simulations, the bonded interactions are often described by effective potential energy functions (analogous to those invoked in conventional particle-based schemes), whereas the nonbonded interactions are described by a free energy functional involving a spatial integral of a free energy density depending on one or more particle density fields. The evaluation of the nonbonded free energy entails discretizing the simulation domain to account for the local densities and perform spatial integration.

A popular approach for discretizing the simulation domain is the imposition of a mesh across it. The local density is described at the level of the mesh cells based on the contributions of the particles participating in them.^29,33^ Mesh-based schemes have been shown to be effective in terms of conserving the proper density and compressibility of bulk systems,^5,33,39^ reproducing the structural features of multicomponent systems,^29^ and describing the interfacial free energies of polymer–solid^40^ and polymer–vacuum^41^ interfaces in conjunction with higher-order corrections, such as the square gradient theory (SG).^42−44^ Because the density is conserved at the cell and not at the particle level, mesh-based approaches can become cumbersome in some cases. For example, when applied to systems with spherical geometry such as droplets and spherical cavities, they may introduce discretization artifacts^40,41^ and induce a limit to the maximum resolution. It should be mentioned, however, that recent years have seen the rise of advanced reciprocal-space approaches that provide superior control over discretization artifacts and resolution limitations.^45,46^

An alternative class of discretization schemes is the so-called meshless approaches. Unlike mesh-based methods that rely on a fixed grid, meshless methods define the domain using the particles themselves. Such meshless discretization schemes are generally more flexible and applicable for describing arbitrary geometries.^27,44,47−53^ While grid-based methods excel in homogeneous systems (and in many cases perform better), meshless methods excel in simulating large deformations and moving boundaries, e.g., during fracture phenomena.^8,9,47^ In this context, the utilization of the so-called kernels allows for estimating the local density at the level of individual particles.^26,54−57^ The density field at each particle is estimated by imposing a weighting kernel, which accounts for the contribution of the particles in the local vicinity. It is well known that the kernels invoked by these schemes suffer from various deficiencies, such as tension instability and artificial clumping,^8,47,57−59^ and boundary issues.^47^ Significant effort has been made to address the aforementioned issues in regard to improved weighting kernels,^50,60−62^ the so-called artificial stress and viscosity,^9,63,64^ staggered meshes and stress-points (stress-particle SPH),^52,65^ the moving least-squares SPH,^58,66^ the particle shifting scheme,^67^ square gradient terms,^13,44,68^ invoking ghost particles,^50,69,70^ and modifying the weighting kernel^27,44,50−53^ at the boundaries. The aforementioned remedies are not crucial in homogeneous systems, as tension instability increases significantly as the system moves far away from equilibrium, in the postfracture regime.^64,71^

Here we develop a meshless discretization scheme for particle-field Brownian dynamics simulations. By following the footsteps of relevant SPH,^26^ SDPD,^68^ and MDPD^13−15^ implementations, the domain discretization is realized by ascribing an effective number density at the position of each particle as a weighted average of mass contributions from the neighboring particles in the close vicinity. Following the discussion on mesh-based and meshless methods, our approach naturally benefits from the advantages we outlined (such as describing complex geometries and large deformations), but may also experience the drawbacks mentioned. To the best of the authors’ knowledge, there are limited (if any) mesoscale simulation frameworks incorporating kernel-based discretization schemes alongside Langevin dynamics in the high friction limit. It is noteworthy that the symmetric nature of interparticle forces^12,25,47,57,62^ enables leveraging optimization techniques from conventional particle simulations, including efficient neighbor lists,^72,73^ optimized minimum image convention,^72^ and parallel simulation paradigms such as MPI and GPU acceleration for improved scalability.^74−76^

The central focus of the article is to assess the numerical stability (NS) of the meshless scheme in terms of reproducing the proper thermodynamics and dynamics of homogeneous samples. The weighting kernel is described with Lucy’s function.^26,54^ For the purpose of testing the scheme, our primary analysis is conducted by utilizing Helfand’s (HFD)^77^ equation of state (EoS), for which the equilibrium density and compressibility can be determined exactly from closed-form expressions. HFD EoS and its extensions (e.g., the Murnaghan EoS^78^) are regularly employed in the literature for bulk samples.^48,79−81^ Note, however, that HFD EoS can approximate any EoS under bulk conditions around a reference density; hence, our analysis is directly transferable to more elaborate EoS for continuous phases. The latter is corroborated through additional investigations on realistic fluids by employing the Sanchez–Lacombe EoS.^82,83^ The performance of the model in inhomogeneous geometries and the effect of the aforementioned remedies on thermodynamics will be explored in a subsequent publication.

NS depends on the choice of model parameters such as monomer mass, coarse-graining degree, thermodynamic conditions, monomeric friction coefficient, time step, and the properties of the weighting kernel. Improper parameter choices can have detrimental effects on the thermodynamic and dynamical properties of the samples and the effect might not be apparent on first inspection. For example, specific parameter combinations may reproduce the correct density but overestimate the compressibility by orders of magnitude.

We invoke a reduced description that takes into account the interrelations of the aforementioned parameters and simplifies our analysis considerably. In particular, we find that NS depends strictly on the reduced reference compressibility of EoS, the ideal number of interacting particles within the range of the kernel, the time step in relation to the friction coefficient, and the reduced external pressure, the latter being relevant when enforcing isobaric conditions. We demonstrate that appropriate parametrization allows for precise restoration of thermodynamics. In practical cases, the accuracy can be further enhanced through a simple renormalization process that involves carrying out an extra simulation. The scheme yields precise dynamics for reasonable parameter choices as well, which can be restored in an exact fashion through a trivial manipulation of the time step and friction coefficient.

We provide a semiempirical relation regarding the upper bound for acceptable time steps, above which the scheme becomes numerically unstable. The relation accounts for the effect of varying the EoS compressibility and friction factor exactly, regardless of the choice of the weighting kernel; the effect of the latter is described in an empirical manner.

The impact of the coarse-graining degree on entropy introduces unexpected side effects in regard to the equilibrium density and compressibility. These effects may lead to unintended complications in some setups. We illustrate the possibility to formulate EoS coefficients by taking account of the coarse-graining degree, ensuring an exact replication of the target density and compressibility. This approach can be extended to more elaborate EoSs.

The article is structured as follows: Section 2.1 provides information regarding the reduced description of the model, Section 2.2 reports the mathematical formulation of the model, Section 2.3 discusses the core assumptions underlying kernels, and Section 2.4 conducts a scaling analysis of the equations of motion, which quantifies the effect of reduced compressibility and friction coefficient on the time step. Section 3.1 displays the capability of the scheme to achieve proper thermodynamics and dynamics across the parameter range considered here, Section 3.2 demonstrates the universal behavior in the reduced description and a renormalization scheme for enhanced performance, and Section 3.3 provides a recipe for canceling the effect of coarse-graining degree on equilibrium thermodynamics. Section 3.4 applies the model to realistic fluids composed of coarse-grained particles. Section 4 concludes the manuscript. The appendices report the density and kernel gradients and demonstrate the equivalence of the derived formula for force with common expressions from the literature. The Supporting Information, which includes refs (39,72,73,84−89), discusses the implementation of the scheme and the contributions of self- and interparticle interactions to force and stress, provides detailed information regarding the simulation protocol, and presents benchmarks for validating the consistency of the reduced description.

## 2 Methods

### 2.1 Model System and Reduced Description

**Figure 1:**
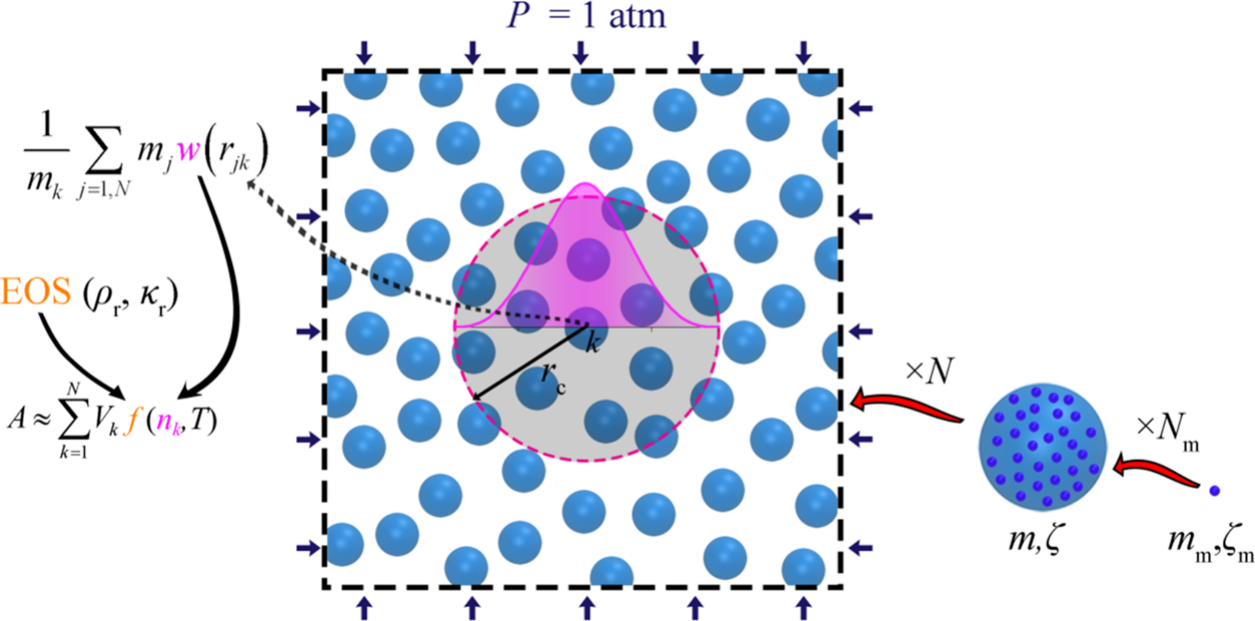
Schematic illustration of a fluid composed of *N* coarse-grained particles. Each particle represents a set of *N*_m_ monomers, each characterized by mass *m*_m_ and monomeric friction coefficient ζ_m_. The system is simulated in the *NPT* ensemble under atmospheric pressure. Particle interactions are governed by an EoS subject to a reference density (ρ_r_) and compressibility (κ_r_). Particle density (*n*_*k*_ = *ρ*_*k*_*/m*_k_) is estimated using a weighting kernel (*w*) that takes into account the influence of neighboring particles within a specified cutoff distance *r*_c_.

Consider a system with *N* coarse-grained (CG) particles occupying volume *V*_sim_ at temperature *T*, each of mass
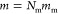
1where *N_m_* is the number of monomers (coarse-graining degree) represented by one particle and *m_m_* is the monomer mass, e.g., see Figure 1. The corresponding average particle density measured over the whole system is
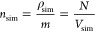
2

The thermodynamics is described by an excess Helmholtz free energy of the form
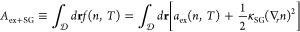
3which is modeled in terms of a functional integral of the excess free energy density (*a*_ex_) in conjunction with a square gradient term^13,42−44,68^ over the system domain (), with *n* = *n*(*r*) being the local number density.

The particles execute position Langevin dynamics in the high friction limit (Brownian motion) subject to the stochastic equation of motion
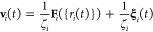
4with **F** being a conservative force, **ξ** a stochastic force satisfying the fluctuation–dissipation theorem, and ζ the friction factor for a particle:
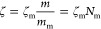
5where ζ_m_ is the monomeric friction coefficient.

Let *n*_r_ = ρ_r_/*m* be a reference particle density and κ_r_ a reference isothermal compressibility. The model parameters will be nondimensionalized in terms of the reference quantities:
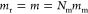
6
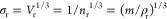
7
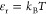
8
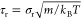
9where *m*_r_ is defined as the particle mass, *σ*_r_ is a characteristic length scale dictated by a reference number density *n*_r_, and *ε*_r_ is the thermal energy. Henceforth, the reduced quantities will be indicated by a tilde above them.

As a consequence of Buckingham’s π-theorem of dimensional analysis,^90^ the reduced description entails that *ñ* = *n*/*n*_r_ = ρ/ρ_r_ = ρ̃ and *ñ*_r_ = *m̃* = *T̃* = 1; hence, the effect of varying κ_r_, ρ_r_, *m*_m_, *N*_m_, and *T* is lumped to the reduced reference isothermal compressibility:
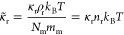
10and the reduced friction coefficient
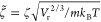
11

The former can be envisioned as the reference compressibility of the sample relative to the compressibility of an ideal gas with particle density *n*_r_ at temperature *T*.

### 2.2 Evaluation of the Excess Helmholtz Free Energy

The discretization of the reduced excess Helmholtz free energy,
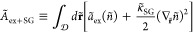
12can be realized by ascribing an effective number density on the particle level as a weighted average of the number of neighboring particles:^12,25,26,57,62^
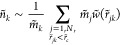
13where *r̃*_*jk*_ = |**r̃**_*jk*_|, **r̃**_*jk*_ = **r̃**_*k*_ – **r̃**_*j*_, *m̃*_*k*_ is the mass of the *k*th particle, and *w̃* is a weighting kernel with a finite support at *r̃*_c_. It is worth mentioning Trofimov et al.’s^14^ weighting scheme that excludes self-interactions (i.e., *j* ≠ *k* in eq 13) and recovers the exact density for homogeneous ideal gas fluids. Subsequently, eq 12 becomes
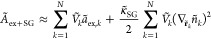
14where *ã*_ex,*k*_≡*ã*_ex_(*ñ*_k_), and *Ṽ*_*k*_≡1/*ñ*_*k*_=*m̃*_*k*_/ρ̃_*k*_ is the volume ascribed to each particle. The discretization of the free energy functional into explicit particle-based contributions aligns with derivations found in relevant DPD implementations.^13,14^ For example, compare eq 14 with eqs 3 and 4 in ref (13), and with eq 16 in ref (14). Note that, even though the “thermodynamic” system volume based on the summation of the particle volumes
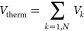
15is not guaranteed to sum to *V*_sim_, in dense enough systems the two volumes are expected to be similar.^26^

The contribution of the excess free energy density to interparticle forces is
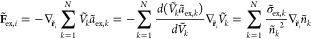
16

The right-hand side is expressed in terms of the particle excess stress (σ̃_ex,*k*_≡*dÃ*_ex.*k*_/*dṼ*_*k*_, *Ã*_ex.*k*_ = *Ṽ*_*k*_*ã*_ex_,_*k*_) and particle density derivative (∇_***r̃***_*i*__*ñ*_*k*_ = −*ñ*_*k*_^2^∇_***r̃***_*i*__*Ṽ*_*k*_). As we demonstrate in Appendix D, eq 16 is equivalent to the symmetric form reported in the literature.^12,25,47,57,62^

The contribution of the SG term to interparticle forces is
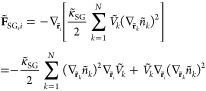
17

By expressing ∇_**r̃**_*i*__*Ṽ*_*k*_ with respect to the density gradient and substituting ∇_**r̃**_*i*__(∇_**r̃**_*k*__*ñ*_*k*_)^2^ from eq 66 in Appendix B we get
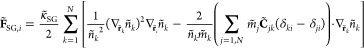
18with **C̃**_*jk*_ being a tensor defined in eq 62, Appendix B. For additional information regarding the density gradients and the kernels, the reader is referred to Appendices A–C.

Owing to the pairwise nature of the interactions, the stress tensor can be determined directly from the forces with the Virial formula.^91^ The implementation of the scheme and the contributions of the self- and interparticle interactions to force and Virial are reported in the Supporting Information S1.

The excess free energy density will be described with the HFD expression:
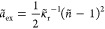
19in terms of the reduced reference compressibility and particle density. The expressions for the corresponding pressure and isothermal compressibility are the following:
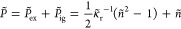
20
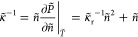
21where the term *ñ* on the right-hand side in eqs 20 and 21 constitutes the ideal gas contribution. It is notable that κ̃_r_ equals the reduced excess compressibility at the reference density (κ̃_r_ = κ̃_ex_(*ñ*_r_)).

The equilibrium density *ñ*_eos_ at a prescribed pressure can be calculated analytically by solving eq 20 for *ñ*:
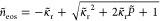
22and the corresponding isothermal compressibility κ̃_eos_ by inputting *ñ*_eos_ to eq 21.

### 2.3 Underlying Assumptions and Kernel Renormalization

Let *g*(*r*) denote the radial distribution function of a sample. The mean number of particles at distance *r* from a reference particle equals, *N*(*r*) = *n*_sim_*g*(*r*). The effective number density of the *k*th particle can be estimated using a mean-field approach based on the following equation:
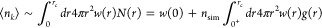
23

Conventionally, the weighting kernel is normalized as follows:^13,14,26,68^
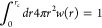
24

By treating the fluids as ideal gases (infinite compressibility, ), by omitting the self-interactions (*w*(0) → 0),^14^ and by taking account of the normalization in eq 24, eq 23 becomes exact:
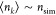
25

Under these ideal gas conditions, the number of interacting particles within the range of the kernel can be determined according to eq 26.
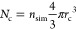
26

**Figure 2:**
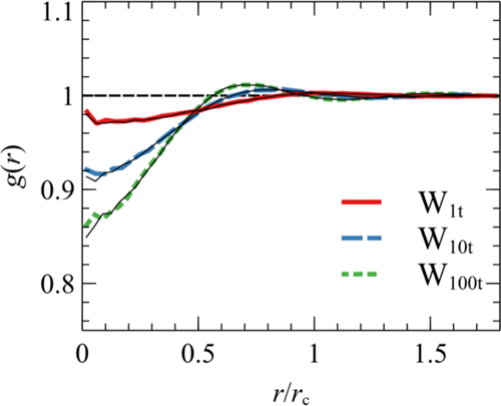
Radial distribution function *g*(*r*) versus *r*/*r*_c_ from systems W_1t_ (solid line, *r*_c_ = 12.2 Å), W_10t_ (dashed line, *r*_c_ = 26.3 Å), and W_100t_ (dotted line, *r*_c_ = 56.8 Å) in Table 2. The thin black lines correspond to results for *C*_w_ = 1. The dashed horizontal line displays the *g*(*r*) of an ideal gas. The compensating factor *C*_w_ from the numerical fitting (from the self-consistent relation in eq 28) equals 0.984 (0.982), 0.994 (0.993), and 0.998 (0.997) for W_1t_, W_10t_, and W_100t_, respectively.

For nonideal gas fluids, the equivalence becomes less accurate. This limitation arises from the *ad hoc* normalization in eq 24, which neglects fluid structure since *g*(*r*) ≠ 1. As discussed in Trofimov et al.,^14^*g*(*r*) features a “correlation hole,” i.e., a region of depleted density caused by the repulsive forces between particles. This effect is visualized by the *g*(*r*) in Figure 2, which reveals the nonuniform structure of nonideal gas fluids and the depletion region that becomes more pronounced with increasing coarse-graining degree. In addition, eq 24 does not take into account the self-interactions *w*(*r* = 0), which partially compensate for the correlation hole in *g*(*r*). These assumptions improve with increasing *r*_c_, where bulk contributions dominate eq 23 (*g*(*r*) ≈ 1, number of interactions scaling as ∼ *r*^3^) and the self-interactions become less important.

The discrepancy can be further suppressed by renormalizing the weighting kernel with a compensating factor *C*_w_, accounting for the self-interactions and fluid structure:
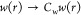
27

In principle, *C*_w_ can be estimated self-consistently in a mean-field manner by applying eqs 25 and 27 to eq 23 and solving for *C*_w_:
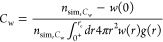
28with *n*_sim,*C*w_ being the mean number density from a simulation carried out with a prescribed value of *C*_w_. Physically, *C*_w_ scales the thermodynamic volume^26^ (eq 15) to match the system’s actual volume. Interestingly, Trofimov et al.^14^ use a similar strategy as in eq 27 to restore the volumetric properties of the fluids, in terms of fitting the density kernel with a linear function accounting for the structure of the fluid.^14,15^

### 2.4 Reduced Units and Numerical Stability (NS)

NS of the nonbonding scheme in terms of properly describing the bulk thermodynamics and kinetics depends on several parameters such as the monomer mass (*m*_m_) and the coarse-graining degree (*N*_m_), the parameters of the EOS (e.g., ρ_r_ and κ_r_ for HFD), the thermodynamic conditions (*T*, *P*), the time step in relation to the friction factor, and the properties of the kernel function.

As a previous grid-based study^41^ reported, finer discretization (lower *N*_m_) leads to a slight increase in residual stress in bulk samples. The increase was minimal (0–4 atm) for κ_SG_ corresponding to realistic surface tension values. Since the SG term has little impact on bulk properties, we will ignore the effect of κ_SG_, i.e., set κ_SG_ = 0 from now on.

The thermodynamics in the reduced description is independent of particle mass, temperature, and density (*m̃* = *T̃* = *ñ*_r_ = 1), and can be described by κ̃_r_. The range of the kernel constitutes a numerical parameter and can be expressed in terms of the ideal number of interacting particles at the reference number density (eq 26):
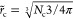
29

The thermodynamics is independent of the friction factor, the latter being a kinetic property.

We can infer the effect of model parameters on the critical time step Δ*t̃*_crit_—below which the scheme is numerically stable—by conducting a scaling analysis on the stochastic differential eq (eq 4) whose reduced form can be discretized (e.g., with Euler’s method) as follows:
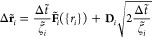
30

The right-hand side involves a stochastic displacement with variance 2Δ*t̃*/ζ̃_*i*_, with the triplet of random variables . By applying the expression for the excess part of the force (eq 16) and the excess particle stress from the EoS (σ̃_ex_=–*P̃*_ex_, from eq 20), eq 30 can be expanded as follows:
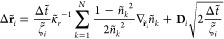
31

We notice that the conservative part of the displacement scales proportionally with 1/κ̃_r_ and Δ*t̃*/ζ̃_*i*_. In addition, as long as Δ*t̃*/ζ̃_*i*_ is constant, the dynamics is unaffected. The part within the summation depends strictly on the properties of the kernel (i.e., *N*_c_ or *r̃*_c_). For fixed *N*_c_, we get a scaling:
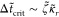
32or, in real units,
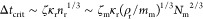
33

We note that Δ*t*_crit_ is *T*-independent and scales sublinearly with the coarse-graining degree (∼*N*_m_^2/3^).

## 3 Results and Discussion

### 3.1 Evaluating the Numerical Stability across a Reduced Parameter Space

The calculations were conducted in the isothermal–isobaric ensemble (*NPT*) by following the simulation protocol reported in Supporting Information S2. Throughout the simulations, ζ_m_, *m*_m_, ρ_r_, *N*, *P*, and *T* were fixed and *N*_c_, *N*_m_, and κ_r_ were varied (see Table 1). Note the correspondence between mass (*m*) and molar mass (*M*), measured in kg/mol: *m* = *M*/(1000*N*_A_) with *N*_A_ being Avogadro’s number. As discussed in Section 2.4, arbitrary combinations of Δ*t*, ζ_m_, *m*_m_, ρ_r_, *N*_m_, and κ_r_ which yield the same κ̃_r_ and Δ*t̃*/ζ̃_*i*_ result in the same reduced sample properties, e.g., see benchmarks in the Supporting Information S3. As our analysis focuses on the bulk properties of the samples, the square gradient term (κ_SG_) is set to zero in this study.

**Table 1:** Simulation Parameters in Real and Reduced Units. Note that ζ_m_, *m*_m_, and ρ_r_ Correspond to the Monomeric Friction Coefficient, Monomer Mass, and Density of High-Density Polyethylene (HDPE) at 450 K^5,92^

kind	parameter	real	reduced
reference	σ_r_	(*N*_m_*m*_m_/ρ_r_)^1/3^	1
	ε_r_	*k*_B_*T*	1
	*m*_r_	*N*_m_*m*_m_	1
	τ_r_	σ_r_(*m*_r_/ε_r_)^0.5^	1
fixed	ζ_m_ [kg/s]	4.15 × 10^–13^	ζ_m_*m*_r_/τ_r_
	*m*_m_ [kg]	14.02658/(1000*N*_A_mol)	1/*N*_m_
	ρ_r_ [kg/m^3^]	766.947	1
	*N*	2000	2000
	*P* [atm]	1	[1 atm] σ_r_^3^/ε_r_
	*T* [K]	450	1
	κ_SG_ [J m^5^]	0	0
variable	*N*_m_	{32, 128, 512}	{32, 128, 512}
	*N*_c_	{32, 64, 128}	{32, 64, 128}
	κ_r_ [GPa^–1^]	{0.1, 1, 10} × 4.8881	{0.1, 1, 10}/*N*_m_

Interestingly, maintaining the system pressure at a nonzero value (e.g., *NPT* simulations) introduces an unexpected side effect in terms of NS, in that the external pressure *P̃* in the reduced description increases proportionally with *N*_m_, i.e., *P̃* = *Pσ*_r_^3^/ε_r_ ∼ *N*_m_. It will be shown that varying *P̃* does have an effect on the NS of the scheme, albeit it is relatively weak in our case. It can, however, become considerable with increasing *P*.

We will explore the NS of the scheme over a broad parameter space in terms of varying *N*_m_ = {32, 128, 512}, *N*_c_ = {32, 64 and 128}, and κ̃_rm_ = {0.1, 1, 10}; the latter is the reference compressibility of the sample relative to the compressibility of an ideal gas with monomer density *n*_m_ at temperature *T*:
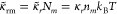
34

NS will be quantified in terms of achieving the equilibrium particle density (*ñ*_sim_) and isothermal compressibility (κ̃_sim_), with respect to their exact values *ñ*_eos_ (eq 22) and κ̃_eos_ (eq 21), respectively. The effect on kinetics will be assessed in terms of comparing the reduced self-diffusion coefficient (*D̃*_sim_) relative to the exact value from Einstein’s model:^93^
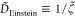
35

**Figure 3:**
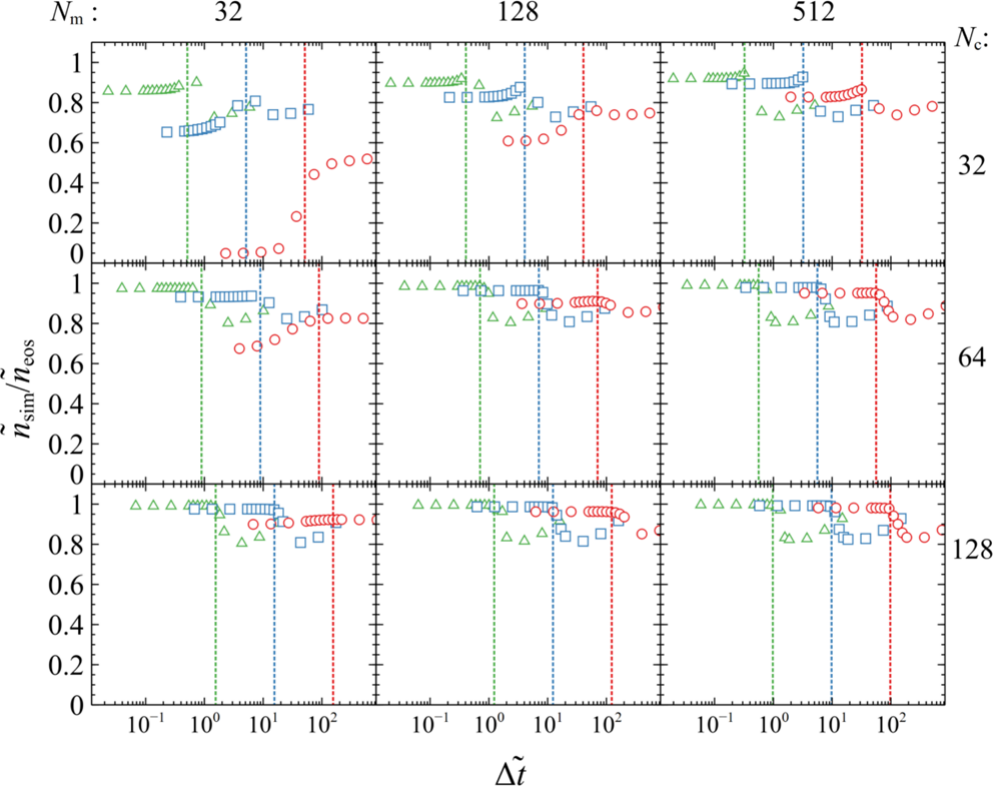
*ñ*_sim_/*ñ*_eos_ versus Δ*t̃*, for *N*_m_ = 32, 128, 512 (from left to right), *N*_c_ = 32, 64, 128 (from top to bottom) for κ̃_rm_ = 0.1 (green, △), 1 (blue, □), and 10 (red, ○). The vertical dotted lines indicate the critical time step from eq 36 for each case.

Figure 3 illustrates the equilibrium particle density as a function of *N*_m_ (left to right), *N*_c_ (top to bottom), and κ̃_rm_ (different symbols) with varying Δ*t̃*. The density plateaus at low Δ*t̃*, whereas, after exceeding a threshold time step, the simulation becomes unstable and density diverges. In terms of reproducing the EoS density, the performance improves with increasing *N*_c_ because the effect of the discretization artifacts in the kernel becomes weak. The situation improves with increasing *N*_m_ and decreasing κ̃_rm_ as well because the sample becomes less compressible (κ̃_r_ decreases in both cases) and can better maintain the target density.

Increasing *N*_m_, κ̃_rm_, and *N*_c_ allows working with larger time steps. By taking into account the scaling of *N*_m_ and κ̃_r_ from eq 32 and optimizing for *N*_c_, we derive the following semiempirical formula:
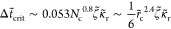
36or
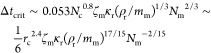
37in real units. In conducting this fit, we have disregarded parameter combinations that result in very poor performance even for small time steps; all cases where *N*_c_ = 32, and the case where *N*_c_ = 64, *N*_m_ = 32, and κ̃_rm_ = 10. Equation 36 yields a reasonable high limit for acceptable time steps as illustrated by the evaluations (vertical dashed lines) in Figure 3.

**Figure 4:**
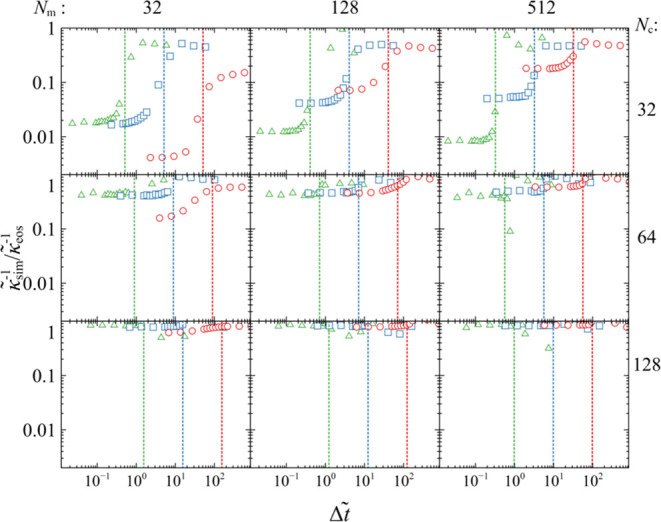
Same as Figure 3 but for κ̃_sim_^–1^*/κ̃*_eos_^–1^.

Figure 4 illustrates numerical evaluations of the simulated isothermal compressibility that was estimated via finite differences by conducting an additional simulation at a slightly higher pressure:
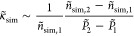
where *P̃*_1_*ε*_r_/σ_r_^3^ = 1 atm and *P̃*_2_*ε*_r_/σ_r_^3^ = 5 atm. Similar to density, the compressibility plateaus (diverges) at short (long) time steps commensurate with Δ*t̃*_crit_ (vertical lines) from eq 36. In terms of reproducing the correct compressibility, the performance is improved with increasing *N*_m_ (left to right) but most importantly with increasing *N*_c_. In particular, setting *N*_c_ to 32 (64) yields a compressibility, which is about 1–2 orders of magnitude (2 times) higher than κ̃_eos_. On the contrary, for *N*_c_ = 128, the compressibility is reproduced quite accurately.

**Figure 5:**
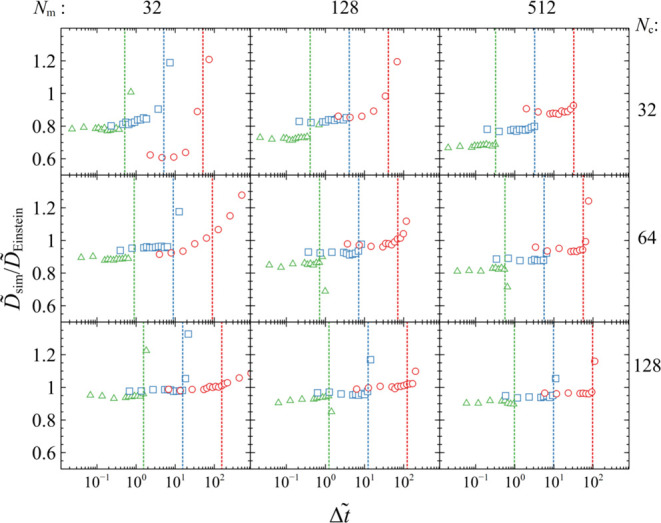
Same as Figure 3 but for *D̃*_sim_/*D̃*_Einstein_.

The effect of the model parameters in terms of achieving the proper dynamics is illustrated in Figure 5, which presents evaluations of *D̃*_sim_/*D̃*_Einstein_ across the parameter space. For low *N*_c_, the diffusion coefficient is significantly lower than the predicted value from Einstein’s model due to perturbations from the intermolecular interactions. With increasing *N*_c_, however, the two become very similar, i.e., see the last row of Figure 5. Excluding cases that result in very low densities, we notice that the correspondence is improved with increasing compressibility because the sample softens and the perturbations become weaker.

### 3.2 Universal Response and Renormalization

**Figure 6:**
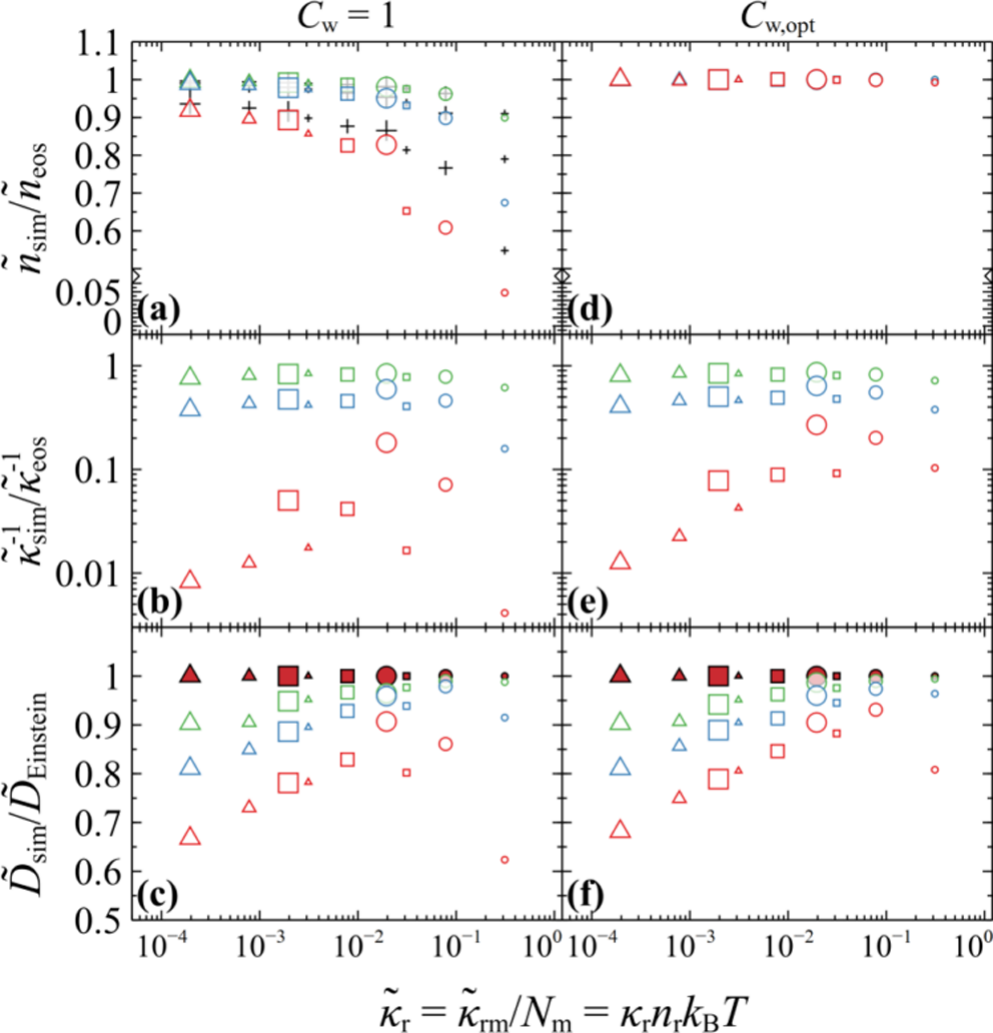
Master plots of converged (a, d) *ñ*_sim_/*ñ*_eos_, (b, e) κ̃_sim_^–1^/κ̃_eos_^–1^, and (c, f) *D̃*_sim_/*D̃*_Einstein_ versus κ̃_r_, for κ̃_rm_ = 0.1 (△), 1 (□), and 10 (○); *N*_m_ = 32 (small), 128 (medium), and 512 (large); and *N*_c_ = 32 (red), 64 (blue), and 128 (green). In the left (right) panels, the evaluations are conducted with the original *C*_w_ = 1 (renormalized, *C*_w,opt_) kernel. The coefficients *C*_w,opt_ are shown with crosses in panel (a). The filled symbols in (c) and (f) illustrate the effect of readjusting the friction factor (eq 39) and time step (eq 40).

The left panels of Figure 6 illustrate master plots of the converged (plateau values for low Δ*t̃*) *ñ*_sim_/*ñ*_eos_, κ̃_sim_^–1^/κ̃_eos_^–1^, and *D̃*_sim_/*D̃*_Einstein_ versus κ̃_r_, across the full range of *N*_c_, *N*_m_, and κ̃_rm_ considered in Figures 3, 4, and 5, respectively.

The properties of each sample depend strictly on *N*_c_ and κ̃_r_, and not on the individual values of κ̃_rm_ and *N*_m_. However, because the simulations are realized in the *NPT* ensemble with *P* = 1 atm, we have to take into account the effect of the *N*_m_-dependent external reduced pressure: *P̃* = *Pσ*_r_^3^/ε_r_ ∼ *N*_m_.

Beginning our inspection with the most numerically stable case (*N*_c_ = 128, green markers in Figure 6a–c), we note that the markers collapse on a single curve. The *ñ*_sim_/*ñ*_eos_ and *κ̃*_sim_^–1^/*κ̃*_eos_^–1^ exhibit similar behavior: they plateau at ∼1 at low κ̃_r_ and drop monotonically at high κ̃_r_. On the contrary, *D̃*_sim_/*D̃*_Einstein_ increases with κ̃_r_ because the sample becomes softer and the Brownian motion becomes less perturbed.

With decreasing *N*_c_, however, there are noticeable deviations from the exact behavior. For example, for *N*_c_ = 32, the (larger) points that correspond to the largest *N*_m_ considered here feature increased *ñ*_sim_, κ̃_sim_^–1^, and *D̃*_sim_ relative to the other points. This happens because, for low *N*_c_, the sample becomes very compressible and as a result the change to the external pressure with increasing *N*_m_ has a significant effect.

It becomes apparent that the assumptions invoked in the definition of the weighting kernels (see Section 2.3) become very approximate in situations where the range of the kernel is short. Nevertheless, by reweighting the kernel, it is possible to enhance its performance in terms of achieving the correct density, or conversely, to bring the “thermodynamic” volume (*V*_therm_, eq 15) closer to the system volume *V*_sim_.

The right-hand side panels of Figure 6d–f illustrate the same master plots but from simulations with renormalized weighting kernels, where the *C*_w_ factor in eq 27 has been optimized with a Newton–Raphson method^94^ to reproduce the correct density. The optimized coefficients in each case are displayed in Figure 6a with crosses. By construction, the simulations with the optimized coefficients yield very accurate density, i.e., *ñ*_sim,*C*w,opt_ ∼ *ñ*_eos_ in Figure 6d.

From a practical viewpoint, it is notable that in cases where *N*_c_ and κ̃^–1^ are not too low, the optimized coefficients are very close to the ratio *ñ*_sim_/*ñ*_eos_ from a simulation with the normal kernel (Figure 6a):
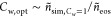
38

This is because *ñ*_sim_/*ñ*_eos_ captures the discrepancy between *V*_therm_ and *V*_sys_, and thus, by multiplying the kernel with *ñ*_sim_/*ñ*_eos_, we can partially negate this effect.

The compressibility of the sample with the renormalized kernel improves as well (e.g., compare panels b and e of Figure 6), albeit it remains still much lower than κ̃_eos_ in cases with low and moderate *N*_c_. It is thus imperative to set *N*_c_ to a high value in applications where a correct description of the compressibility is important.

*D̃*_sim_ improves slightly in situations where the kernel is renormalized (e.g., compare panels c and f of Figure 6). However, this is of minor importance, since it is trivial to restore the exact (or any arbitrary) value. According to Einstein’s model (eq 35), scaling ζ̃ by an arbitrary constant *C*_ζ_ will scale *D̃*_Einstein_ by 1/*C*_ζ_. In addition, scaling Δ*t̃* and ζ̃ by the same amount does not affect NS (see discussion in Section 2.4 and eq 31). By taking the above into account, we can reproduce *D̃*_Einstein_ exactly by rescaling Δ*t̃* and ζ̃ as follows:
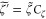
39
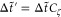
40where
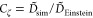
41

In doing so, the diffusivity is reproduced exactly, regardless of whether the kernel is renormalized (see filled markers in Figure 6c,f).

### 3.3 Canceling the Effect of Coarse Graining on Thermodynamics

Let ρ_t_ and κ_t_ be the target mass density and compressibility of a system with coarse-grained particles, each one having a mass *N*_m_*m*_m_ at temperature *T* and pressure *P*. By expressing the free energy density with the HFD model, eq 19, we get the following equations for pressure and compressibility:
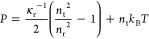
42
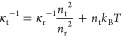
43

Because the ideal gas contribution (right-hand side of eqs 42 and 43) is a function of the coarse-graining degree (*n*_t_ = ρ_t_/*N*_m_*m*_m_ ∼ *N*_m_^–1^), the equilibrium density and compressibility vary with *N*_m_. In many applications, this may be an unwanted behavior.

Conveniently, we can calculate analytically the reference density (ρ_r_ = *n*_r_*N*_m_*m*_m_) and compressibility (κ_r_), which reproduce ρ_t_ and κ_t_ as a function of *T*, *P*, and *N*_m_. First, we solve eq 43 for κ_r_:
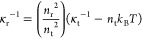
44

By substituting κ_r_ from eq 44 into eq 42, we get the following expression for *n*_r_:
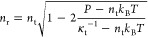
45

Finally, we derive the expression for κ_r_ by substituting *n*_r_ from eq 45 to eq 44:
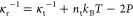
46

The resulting *n*_r_ and κ_r_ allow to reproduce the target density and compressibility of the system regardless of the choice of the coarse-graining degree.

### 3.4 Application to Real Fluids Composed of Coarse-Grained Particles

In this section, we apply the nonbonding scheme for realistic fluids composed of coarse-grained particles, employing various EoS.

**Figure 7:**
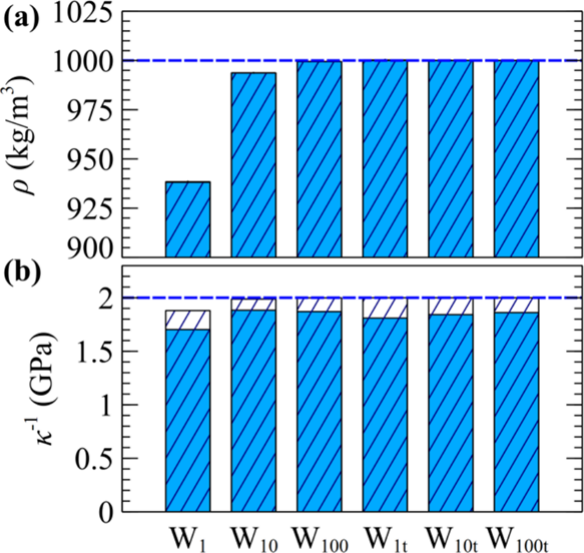
(a) Density and (b) inverse compressibility of coarse-grained water particles using the parameters listed in Table 2. The colored bars illustrate the simulated quantities, while the textured ones indicate the exact values determined by EoS, i.e., ρ_eos_ and κ_eos_ from Table 2. The dashed lines correspond to the experimental density and inverse compressibility.

Figure 7 presents the equilibrium density and inverse compressibility of coarse-grained water particles at *T* = 277 K and *P* = 1 atm, considering different degrees of coarse graining (*N*_m_). The experimental density and compressibility under these conditions are ρ_exp_ = 1000 kg/m^3^ and κ_exp_ ∼ 0.5 GPa^–1^,^95^ respectively. In addition, the self-diffusion coefficient of water molecules is *D*_exp_ = 1.261 × 10^–9^ m^2^/s.^96^ The calculations are conducted in the *NPT* ensemble using HFD EoS and renormalized weighting kernels with parameters from Table 2.

**Table 2:** Simulation Parameters for a System with *N* = 3000 Water Particles Using HFD EoS at *T* = 277 K and *P* = 1 atm^a^

system	*ρ*_r_ (kg/m^3^)	*κ*_r_ (GPa^–1^)	*N*_m_	*ρ*_eos_ (kg/m^3^)	*κ*_eos_ (GPa^–1^)	*C*_w_	*C*_ζ_
W_1_	1000.00	0.500	1	938.2	0.5318	0.984	0.981
W_10_	1000.00	0.500	10	993.7	0.5031	0.994	0.966
W_100_	1000.00	0.500	100	999.4	0.5003	0.998	0.950
W_1t_	1066.05	0.470	1	1000.0	0.5000	0.984	0.980
W_10t_	1006.36	0.497	10	1000.0	0.5000	0.994	0.966
W_100t_	1000.59	0.500	100	1000.0	0.5000	0.998	0.946

a In All Cases, *N*_c_ = 256, *M*_m_ = 18.01528 g/mol, ζ_m_ = *k*_B_*T*/*D*_exp_ = 3.0328 × 10^–12^ kg/s, κ̃_r_*N*_m_ ∼ 0.064, and Δ*t̃* = 0.1Δ*t̃*_crit_.

The renormalized kernels reproduce the EoS density, as indicated by the filled and textured bars in Figure 7a, which are indistinguishable. On the contrary, the inverse compressibility is slightly lower than its exact value κ_eos_^–1^. The optimal *C*_w_ for each case is in good match with its estimation from the mean-field formula in eq 28 (see the caption of Figure 2). The effect of *C*_w_ on *g*(*r*) is negligible (compare the colored lines with the thin black lines in Figure 2). In addition, the renormalized friction factor reproduces the experimental diffusion coefficient exactly.

Cases W_1_, W_10_, and W_100_ in Figure 7 (where the subscript in this notation denotes *N*_m_) illustrate a naive approach where the EoS coefficients are set to experimental density (ρ_r_ = ρ_exp_) and compressibility (κ_r_ = κ_exp_). For *N*_m_ = 1 (system W_1_), the density is reproduced poorly due to the contribution of the ideal gas term (see discussion in Section 3.3). As *N*_m_ increases, however, the discrepancy progressively decreases. In cases W_1t_, W_10t_, and W_100t_, the coefficients ρ_r_ and κ_r_ have been determined from eqs 45 and 46, respectively, ensuring equality between EoS density and compressibility with their experimental counterparts.

**Figure 8:**
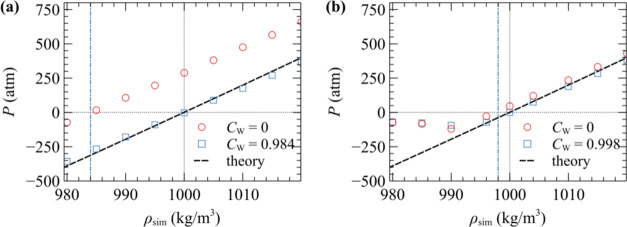
Pressure as a function of density for systems (a) W_1t_ and (b) W_100t_ from Table 2, with (□) and without (○, *C*_w_ = 1) kernel renormalization. These simulations were conducted in the canonical ensemble (*NVT*). The black dashed line displays the theoretical prediction based on eq 42. The blue dot-dashed line denotes the densities *C*_w_ρ_sim,1 atm_ equal to (a) 984 kg/m^3^ and (b) 998 kg/m^3^. The dotted lines are guides to the eye.

Figure 8 illustrates the impact of density variation with pressure for systems W_1t_ and W_100t_, comparing approaches with and without kernel renormalization (*C*_W_ = 1). The renormalized kernels lead to volumetric properties that closely match the analytical predictions from EoS in eq 42. Notably, the bulk density at 1 atm is reproduced perfectly, while the pressure–density slope is in good agreement, albeit slightly weaker due to the slightly higher compressibility (see Figure 7b).

Conversely, evaluations using non-normalized kernels exhibit a significant deviation from the analytical predictions, with this discrepancy becoming more pronounced with decreasing *N*_m_. Intuitively, increasing *N*_m_ should hinder the accuracy of the model, as it makes the correlation hole in the *g*(*r*) more pronounced (e.g., as shown in Figure 2). However, the self-interactions become more effective at compensating for the correlation hole with increasing *N*_m_, thus leading to improved model behavior and compensating factors closer to unity. The dot-dashed line in Figure 8 schematically depicts the relationship between the density of the sample at *P* = 1 atm using the non-normalized kernel and the optimal *C*_w_ for each case; according to eq 38, the optimal *C*_w_ is close to the ratio of ρ_sim_(*C*_w_ = 1)/ρ_eos_. As shown in Figure 8b, the sample with *N*_m_ = 100 cannot withstand large negative pressures, indicating that susceptibility to cavitation increases with the coarse-graining degree.

**Figure 9:**
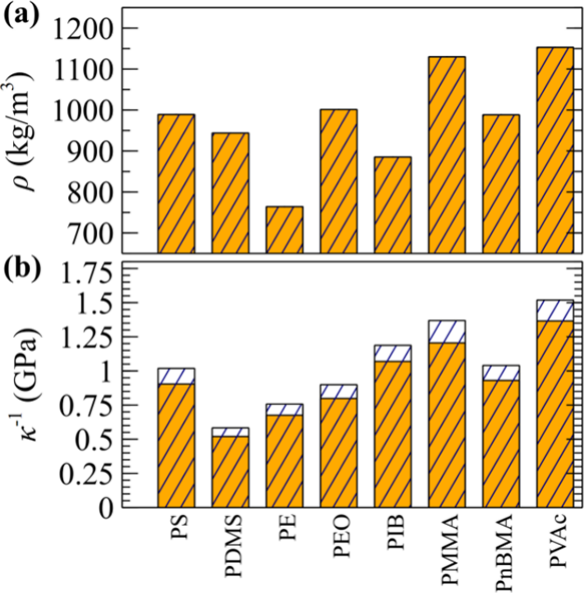
Same as Figure 7 using the parameters listed in Table 3.

Figure 9 showcases applications in homogeneous polymer melts described by the Sanchez–Lacombe EoS^82,83^ with excess pressure:
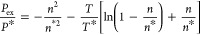
47and excess isothermal compressibility
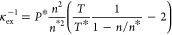
48with parameters the characteristic temperature (T*), pressure (P*), and density (ρ* = mn*). For simplicity, the particle molar mass is set to *M* = 1000 g/mol, assumed to be equal to the polymer molar mass. Note that the effect of chain length is naturally accounted for by the Sanchez–Lacombe EoS through the ideal gas term. The latter is typically expressed as *P*_ig_ = P*ρT/(ρ*T*r), with *r* = P*M/(ρ*RT*) being the number of lattice sites occupied by the polymer chain.

For each case, the reference density (ρ_r_ = *mn*_r_) is determined by solving eq 47 for *n* at zero pressure. Subsequently, the reference compressibility is set to κ_r_ = κ_ex_(*n*_r_). Similar to previous cases, the kernel is renormalized through a single simulation, resulting in a simulated density that aligns perfectly with ρ_eos_, i.e., the filled and textured bars in Figure 9a are indistinguishable. Moreover, the simulated compressibility is slightly underestimated, albeit the effect can be suppressed by further expanding the range of the kernel.

In many practical scenarios, it is convenient to model the polymer chains in terms of multiple particles connected by entropic springs. To achieve the correct scaling of dynamics with chain size and reproduce the experimental viscoelastic properties,^5,33,98^ the scheme should be employed in conjunction with a model that accounts for the effect of entanglements.^34,35^ Various models, such as TWENTANGLEMENT,^99^ slip-link,^36,37^ and slip-spring^5,33,38,100^ models developed in the literature, address this aspect effectively Table 3.

**Table 3:** Simulation Parameters for a System with *N* = 3000 Polymer Particles Using the Sanchez–Lacombe EoS^83^ at *P* = 1 atm^a^

system	T* (K)	P* (MPa)	ρ* (kg/m^3^)	*T* (K)	*ρ*_r_ (kg/m^3^)	*κ*_r_ (GPa^–1^)	*ρ*_eos_ (kg/m^3^)	*κ*_eos_ (GPa^–1^)	*C*_w_	*κ̃*_r_
PDMS^83^	476	302	1104	320	947.6	1.64	943.8	1.71	0.993	0.0041
PVAc^83^	590	509	1283	340	1155.4	0.64	1153.0	0.66	0.993	0.0021
PnBMA^83^	627	431	1125	390	991.2	0.93	988.3	0.96	0.993	0.0030
PIB^83^	643	354	974	354	887.2	0.81	885.4	0.84	0.993	0.0021
PE^83^	649	425	904	450	766.9	1.27	764.2	1.32	0.994	0.0036
PMMA^83^	696	503	1269	414	1132.9	0.70	1129.8	0.73	0.994	0.0027
PS^83^	735	357	1105	428	992.3	0.94	989.0	0.98	0.994	0.0033
PEO^97^	656	492	1180	450	1005.2	1.06	1001.3	1.11	0.994	0.0040

a In All Cases, *N*_c_ = 256, *M* = 1000.0 g/mol, and Δ*t̃* = 0.1Δ*t̃*_crit_.

## 4 Conclusions

The article develops a meshless discretization scheme for particle-field Brownian dynamics simulations. The density is ascribed on the particle level in terms of imposing a weighting kernel with a finite support (*r*_c_). The free energy of the system is described by a free energy density from an EoS in conjunction with a square gradient (SG) term.

The contributions of the EoS and the SG terms to force and stress are derived analytically by differentiating the free energy. We demonstrate that the resulting expression for the EoS contribution to force is equivalent to the common expression used in the literature.

The focus of the article is on determining the numerical stability (NS) of the scheme in bulk conditions. In doing so, we primarily invoke Helfand’s (HFD) EoS with parameters the reference density and compressibility, and we drop the SG term. HFD EoS can serve as an approximation for any EOS under bulk conditions around a reference density. Our analysis is directly applicable to more sophisticated EOS and this has been corroborated here by simulations of realistic fluids utilizing the Sanchez–Lacombe EoS.

The NS of the scheme depends on several parameters, including the monomer mass and coarse-graining degree, the coefficients of the EoS, the thermodynamic conditions (*P*, *T*), the time step, the friction factor, and the properties of the weighting kernel. To take into account the interrelations of the aforementioned parameters to the NS, we invoke a reduced description of the model, which simplifies the analysis considerably.

We find that the outcome of the simulations depends strictly on the reduced reference compressibility (κ̃_r_), the ratio of the friction coefficient to the time step (ζ̃/Δ*t̃*), the range of the kernel function (*r̃*_c_), and the reduced external pressure (*P̃*), the latter being relevant in isothermal–isobaric statistical ensembles.

NS is assessed in terms of reproducing the equilibrium density and compressibility dictated by EoS and the self-diffusion coefficient from Einstein’s model.^93^ For large enough *r̃*_c_, the aforementioned properties are reproduced very accurately. With decreasing kernel range, on the other hand, we observe significant discrepancies because the assumptions invoked in the definition of the kernels become inaccurate.

We develop a scheme for improving the aforementioned deficiencies in terms of renormalizing the weighting kernels. The optimal renormalization of the kernel requires conducting simulations in the *NPT* ensemble, to assess the discrepancy of the density (*ñ*_sim_/*ñ*_eos_) and diffusion coefficient (*D̃*_sim_/*D̃*_Einstein_) relative to their exact values. In practical cases with sufficiently high *r̃*_c_, the density can be restored by multiplying the kernel with *ñ*_sim_/*ñ*_eos_ from a single simulation. In addition, given that *D̃*_Einstein_ ≡ 1/ζ̃ and that the dynamics depends on ζ̃/Δ*t̃* and not on the individual values of ζ̃ and Δ*t̃*, it is trivial to restore the exact diffusion coefficient just by multiplying ζ̃ and Δ*t̃* with *D̃*_sim_/*D̃*_Einstein_. Restoring the compressibility exactly is, however, not trivial; therefore, in applications that necessitate high accuracy, the usage of kernels with long enough range is advised.

The parameters of the model have a strong effect on the choice of acceptable time steps. The effect of κ̃_r_ and ζ̃ on the time step was determined exactly by conducting a scaling analysis on the equations of motion. Even though it is not straightforward to assess the effect of *r̃*_c_ analytically, the latter effect was fitted to a semiempirical formula, eqs 36 and 37, which yields very reasonable estimates regarding the upper bound of acceptable time steps.

The equilibrium density and compressibility depend on the reference density and compressibility of the EoS, the thermodynamic conditions (*P*, *T*), and the coarse-graining degree (*N*_m_). In many applications, the latter introduces undesirable side effects but, as we demonstrate, it is possible to derive analytically *N*_m_-dependent reference densities and compressibilities that reproduce the target equilibrium density and compressibility exactly.

As will be demonstrated in a future publication, the discretization scheme is compatible with the slip-spring BD/kMC model developed by the authors, which has been applied for investigating the rheology of linear^5,33,101^ and branched^102^ high molar mass polymer chains, the elastic properties of rubber materials,^39,98^ as well as interfaces between molten polymers and gases^41^ or solids.^40^

Future directions of this study include the application of the kernel-based discretization scheme to mesoscopic polymer/solid and polymer/polymer interfaces of entangled, high molar mass polymer chains under quiescent and flow conditions, the assessment of the capability of the scheme to predict equilibrium morphologies and interfacial free energies in conjunction with the square gradient theory, and applications in material fracture.

## Appendix A Vector Gradients

Let **r**_*jk*_ = **r**_*k*_ – **r**_*j*_ be a vector pointing from **r**_*j*_ to **r**_*k*_. The length of the vector is denoted with , and the unit vector with **r̂**_*jk*_=**r**_*jk*_/*r*_*jk*_. The gradient of the vector length *r*_*jk*_ with respect to a position vector **r**_*i*_ is

49

with δ_*ij*_ being the Kronecker delta function; δ_*ij*_ = 1 if *i* = *j* and δ_*ij*_ = 0 is *i* ≠ *j*. The partial derivative of a vector **r**_*jk*_ with respect to the component α_*i*_ ∈ (*x*,*y*,*z*) of a position vector **r**_*i*_ is

50

The partial derivative of a component α̂_*jk*_ of a unit vector **r̂**_*jk*_ with respect to the component β_*i*_ of a position vector **r**_*i*_ is

51

The gradient of the unit vector **r̂**_*jk*_ with respect to a position vector **r**_*i*_ yields the Jacobian:

52

By evaluating the partial derivatives of the unit vector components with eq 51, we derive the compact form:

53

with **r̂**_*jk*_⊗**r̂**_*jk*_ being a dyadic tensor and **I**_3_ being the unit second-order tensor in three dimensions.

## Appendix B Density Gradients

### Gradient of the Number Density

The gradient of the number density at the *k*th particle with respect to the position vector of the *i*th particle (eq 13) is evaluated in conjunction with eq 49 as follows:

54

For *i* = *k*:

55

For *i* ≠ *k*:

56

Note the relations between ∇_**r**_*k*__*n*_*k*_ and ∇_**r**_*j*__*n*_*k*_:

57

and ∇_**r**_*i*__*n*_*k*_ and ∇_**r**_*k*__*n*_*i*_, for *k* ≠ *i*:

58

Equation 58 is a demonstration of global momentum conservation.^58^

### Double Mixed Gradient of Number Density

The double mixed gradient of the number density can be determined by taking the gradient of eq 54:

59

By substituting ∇_**r**_*i*__**r̂**_*jk*_ from eq 53 and ∇_**r**_*i*__*r*_*jk*_ from eq 49 we get

60

or in a more compact form:

61

where

62

63

64

### Gradient of the Square Gradient

The gradient of the square gradient can be determined as follows:

65

By substituting eq 61, we derive the general expression:

66

For *l* = *k*:

67

For *l* ≠ *k*:

68

## Appendix C Lucy Weighting Kernel and Derivatives

Lucy’s weighting kernel is illustrated in eq 69.

69

The first and second derivatives are the following:

70

71

The expressions of *A*_*jk*_ and *B*_*jk*_ for calculating **C**_*ij*_ in eq 62 are the following:

72

73

## Appendix D Equivalence of Force Expression with the Symmetric Form

Let us first rewrite eq 16 in real units:

74

The first (second) term describes pairwise (self) interactions. By expanding the first density derivatives (eqs 55 and 56) we get

75

or

76

which is the expression commonly used in the literature.^12,25,47,57,62^ Note that the summation excludes the reference particle,^25^ even though it might not always be explicitly stated.

## Supporting Information Available

(S1) Implementation of the kernel-based discretization scheme; (S2) Simulation protocol; (S3) Validating the internal consistency of the reduced description. (PDF)
