# Supplementary material for: Development of a Meshless Kernel-Based Scheme for Particle-Field Brownian Dynamics Simulations
Source: J Phys Chem B. 2024 Jul 10;128(28):6907–21. doi: 10.1021/acs.jpcb.4c01441 (PMC11264276; doi:10.1021/acs.jpcb.4c01441)
Supplement: Supplementary file 1 — jp4c01441_si_001.pdf [file jp4c01441_si_001.pdf]

Supporting Information: Development of a Meshless Kernel-Based Scheme for Particle-Field Brownian Dynamics Simulations

Aristotelis P. Sgouros, Doros N. Theodorou*

School of Chemical Engineering, National Technical University of Athens (NTUA),

GR-15780 Athens, Greece.

to whom correspondence should be addressed: arissgouros@gmail.com, Tel. +30 210 772 3216

Table of Contents

S1. IMPLEMENTATION OF THE KERNEL-BASED DISCRETIZATION SCHEME 3

S2. SIMULATION PROTOCOL 7

S3. VALIDATING THE INTERNAL CONSISTENCY OF THE REDUCED DESCRIPTION 11

REFERENCES 13

S1. Implementation of the kernel-based discretization scheme

For the convenience of the reader, some equations from the main text are reproduced here in real units, in order to render the analysis self-contained.

The excess free energy of the system can be estimated in terms of the density kernels according to eq S1,

$$A_{\mathrm{ex+SG}} \approx \sum_{k=1}^{N} V_k a_{\mathrm{ex},k} + \frac{\kappa_{\mathrm{SG}}}{2} \sum_{k=1}^{N} V_k \left(\nabla_{\mathbf{r}_k} n_k \right)^2 \tag{S1}$$

where the density gradient can be determined as:

$$\nabla_{\mathbf{r}_i} n_k = \frac{1}{m_k} \sum_{j=1,N} m_j \left. \frac{\partial w}{\partial r} \right|_{r=r_{jk}} \nabla_{\mathbf{r}_i} r_{jk} = \frac{1}{m_k} \sum_{j=1,N} m_j \left. \frac{\partial w}{\partial r} \right|_{r=r_{jk}} \hat{\mathbf{r}}_{jk} \left(\delta_{ki} - \delta_{ji} \right) \tag{S2}$$

Note that in the special case $k \neq i$:

$$\nabla_{\mathbf{r}_i} n_k = -\frac{m_i^2}{m_k^2} \nabla_{\mathbf{r}_k} n_i, \quad k \neq i \tag{S3}$$

The excess and square gradient (SG) parts of the force are the following:

$$\begin{aligned} \mathbf{F}_i &= \mathbf{F}_{\mathrm{ex},i} + \mathbf{F}_{\mathrm{SG},i} \\ &= \sum_{k=1}^{N} \frac{\sigma_{\mathrm{ex},k}}{n_k^2} \nabla_{\mathbf{r}_i} n_k + \frac{\kappa_{\mathrm{SG}}}{2} \sum_{k=1}^{N} \left[\frac{1}{n_k^2} \left(\nabla_{\mathbf{r}_k} n_k \right)^2 \nabla_{\mathbf{r}_i} n_k - \frac{2}{n_k m_k} \left(\sum_{j=1,N} m_j \mathbf{C}_{jk} \left(\delta_{ki} - \delta_{ji} \right) \right) \cdot \nabla_{\mathbf{r}_k} n_k \right] \end{aligned} \tag{S4}$$

where

$$\mathbf{C}_{jk} = A_{jk} \mathbf{I}_3 + B_{jk} \mathbf{r}_{jk} \otimes \mathbf{r}_{jk} \tag{S5}$$

and

$$A_{jk} = \frac{1}{r_{jk}} \left. \frac{\partial w}{\partial r} \right|_{r=r_{jk}} \tag{S6}$$

$$B_{jk} = \frac{1}{r_{jk}{}^2}\left(\left.\frac{\partial^2 w}{\partial r^2}\right|_{r=r_{jk}} - \frac{1}{r_{jk}} \left.\frac{\partial w}{\partial r}\right|_{r=r_{jk}} \right) \quad \text{S7}$$

By merging the two summations over k and by substituting $n_k \rightarrow 1/V_k$, eq S4 becomes:

$$\mathbf{F}_i = \sum_{k=1}^{N} V_k{}^2 \left[\sigma_{\mathrm{ex},k} + \frac{\kappa_{\mathrm{SG}}}{2} \left(\nabla_{\mathbf{r}_k} n_k \right)^2 \right] \nabla_{\mathbf{r}_i} n_k - \kappa_{\mathrm{SG}} \frac{V_k}{m_k} \left(\sum_{j=1,N} m_j \mathbf{C}_{jk} \left(\delta_{ki} - \delta_{ji} \right) \right) \cdot \nabla_{\mathbf{r}_k} n_k \quad \text{S8}$$

or in a more compact form:

$$\mathbf{F}_i = \sum_{k=1}^{N} g_k \nabla_{\mathbf{r}_i} n_k - \kappa_{\mathrm{SG}} \frac{V_k}{m_k} \left(\sum_{j=1,N} m_j \mathbf{C}_{jk} \left(\delta_{ki} - \delta_{ji} \right) \right) \cdot \nabla_{\mathbf{r}_k} n_k \quad \text{S9}$$

where

$$g_k = V_k{}^2 \left(\sigma_{\mathrm{ex},k} + \frac{\kappa_{\mathrm{SG}}}{2} \left(\nabla_{\mathbf{r}_k} n_k \right)^2 \right) \quad \text{S10}$$

We can separate the force exerted on the ith particle into inter-particle (int) and self-particle (self) contributions from the first and second terms of eq S9 as follows:

$$\mathbf{F}_i = \sum_{\substack{k=1 \\ i \neq k}}^{N} \mathbf{F}_{k \rightarrow i}^{\mathrm{g,int}} + \mathbf{F}_{i \rightarrow i}^{\mathrm{g,self}} + \sum_{\substack{k=1 \\ i \neq k}}^{N} \mathbf{F}_{k \rightarrow i}^{\mathrm{SG,int}} + \mathbf{F}_{i \rightarrow i}^{\mathrm{SG,self}} \quad \text{S11}$$

where

$$\mathbf{F}_{k \rightarrow i}^{\mathrm{g,int}} = g_k \nabla_{\mathbf{r}_i} n_k ,\ i \neq k \quad \text{S12}$$

$$\mathbf{F}_{i \rightarrow i}^{\mathrm{g,self}} = g_i \nabla_{\mathbf{r}_i} n_i \quad \text{S13}$$

$$\mathbf{F}_{k \rightarrow i}^{\mathrm{SG,int}} = V_k \kappa_{\mathrm{SG}} \frac{m_i}{m_k} \mathbf{C}_{ik} \cdot \nabla_{\mathbf{r}_k} n_k ,\ i \neq k \quad \text{S14}$$

$$\mathbf{F}_{i \rightarrow i}^{\mathrm{SG,self}} = -V_i \kappa_{\mathrm{SG}} \frac{1}{m_i} \left(\sum_{j=1,N} m_j \mathbf{C}_{ji} \right) \cdot \nabla_{\mathbf{r}_i} n_i \quad \text{S15}$$

Since $\mathbf{C}_{ji} = \mathbf{C}_{ij}$, eq S15 is related to eq S14 as follows:

$$\mathbf{F}_{i\to i}^{\mathrm{SG,self}} = -V_i \kappa_{\mathrm{SG}} \frac{1}{m_i}\left(\sum_{j=1,N} m_j \mathbf{C}_{ji}\right)\cdot\nabla_{\mathbf{r}_i} n_i = -\sum_{j=1,N} \mathbf{F}_{i\to j}^{\mathrm{SG,int}} \qquad \text{S16}$$

where, in eq S14, k and i, have been swapped with i and j, respectively. This relation allows estimating the inter- and the self-particle contributions of the gradient term in the same loop.

The following section depicts a pseudo code for the evaluation of energy, force and stress. For compactness we introduce the quantities:

$$m_{ij} = m_i / m_j \qquad \text{S17}$$

$$\mathbf{w}'_{ij} = -\partial w/\partial r\big|_{r=r_{ij}} \hat{\mathbf{r}}_{ij} \qquad \text{S18}$$

- Compute the "inter" part of the particle densities n_k and the total $\nabla_{\mathbf{r}_k} n_k$:

    for k = 1, N:

        for $j = k + 1$, N:

            Calculate r_{jk}

            if ($r_{jk} < r_{\mathrm{c}}$):

                $n_k \to n_k + m_{jk} w(r_{jk})$

                $n_j \to n_j + m_{kj} w(r_{jk})$

                Calculate $\mathbf{w}'_{jk}$ (no need to evaluate $\mathbf{w}'_{kj}$, i.e., compare with eq S3)

                $\nabla_{\mathbf{r}_k} n_k \to \nabla_{\mathbf{r}_k} n_k - m_{jk}\mathbf{w}'_{jk}$

                $\nabla_{\mathbf{r}_j} n_j \to \nabla_{\mathbf{r}_j} n_j + m_{kj}\mathbf{w}'_{jk}$

- Compute the remaining "self" contributions to n_k, and the g_k and free energy:

    for k = 1, N:

        $n_k \to n_k + w(0)$

        $V_k = 1/n_k$

        Calculate the excess free energy density, $a_{\mathrm{ex},k}$

        Calculate the excess stress, $\sigma_{\mathrm{ex},k}$

        Calculate the square gradient, $\left(\nabla_{\mathbf{r}_k} n_k\right)^2$

Calculate g_k from eq S10

Calculate the free energy (eq S1)

- Calculate the particle force and Virial:

for $i = 1, N$:

for $k = i + 1, N$:

Calculate r_{ik}

if ($r_{ik} < r_c$):

Calculate $\mathbf{w}'_{ik}$ (no need to evaluate $\mathbf{w}'_{kj}$, i.e., compare with eq S3)

$$\mathbf{F}_{k\to i}^{\mathrm{g,int}} = g_k m_{ik} \mathbf{w}'_{ik} \quad \text{(eq S12)}$$

$$\mathbf{F}_{i\to k}^{\mathrm{g,int}} = -g_i m_{ki} \mathbf{w}'_{ik} \quad \text{(eq S12)}$$

Calculate $\mathbf{C}_{ik}$, A_{ik} and B_{ik} from eqs S5-S7; note that $\mathbf{C}_{ik} = \mathbf{C}_{ki}$.

$$\mathbf{F}_{k\to i}^{\mathrm{SG,int}} = V_k \kappa_{\mathrm{SG}} m_{ik} \mathbf{C}_{ik} \cdot \nabla_{\mathbf{r}_k} n_k$$

$$\mathbf{F}_{i\to k}^{\mathrm{SG,int}} = V_i \kappa_{\mathrm{SG}} m_{ki} \mathbf{C}_{ik} \cdot \nabla_{\mathbf{r}_i} n_i$$

$$\mathbf{F}_{i\to i,\,\mathrm{from}\,k}^{\mathrm{SG,self}} = -\mathbf{F}_{i\to k}^{\mathrm{SG,int}} \quad \text{(eq S16)}$$

$$\mathbf{F}_{k\to k,\,\mathrm{from}\,i}^{\mathrm{SG,self}} = -\mathbf{F}_{k\to i}^{\mathrm{SG,int}} \quad \text{(eq S16)}$$

Add the contributions to the per-particle force

$$\mathbf{F}_i \to \mathbf{F}_i + \mathbf{F}_{k\to i}^{\mathrm{g,int}} + \mathbf{F}_{k\to i}^{\mathrm{SG,int}} + \mathbf{F}_{i\to i,\,\mathrm{from}\,k}^{\mathrm{SG,self}}$$

$$\mathbf{F}_k \to \mathbf{F}_k + \mathbf{F}_{i\to k}^{\mathrm{g,int}} + \mathbf{F}_{i\to k}^{\mathrm{SG,int}} + \mathbf{F}_{k\to k,\,\mathrm{from}\,i}^{\mathrm{SG,self}}$$

Add the contributions to the Virial from $\mathbf{F}_{k\to i}^{\mathrm{gi}}$, $\mathbf{F}_{i\to k}^{\mathrm{g,int}}$, $\mathbf{F}_{k\to i}^{\mathrm{SG,int}}$ and $\mathbf{F}_{i\to k}^{\mathrm{SG,int}}$.

Note: the self-interaction terms $\mathbf{F}_{i\to i,\,\mathrm{from}\,k}^{\mathrm{SG,self}}$ and $\mathbf{F}_{k\to k,\,\mathrm{from}\,i}^{\mathrm{SG,self}}$ do not contribute to the Virial.

- Calculate the remaining self-contributions to force:

for $i = 1, N$:

$$\mathbf{F}_{i\to i}^{\mathrm{g,self}} = g_i \nabla_{\mathbf{r}_i} n_i$$

$$\mathbf{F}_i \to \mathbf{F}_i + \mathbf{F}_{i\to i}^{\mathrm{g,self}}$$

Note: the self-interactions do not contribute to the Virial.

The aforementioned scheme has been optimized by implementing efficient neighbor lists, where only 14 of the 27 surrounding cells are considered at each time. In addition, an optimized *minimum image* convention is employed here, where the cell-cell distances were precomputed.[S1,S2]

The consistency between the energy and the force has been validated by comparing the results from the analytical expressions with finite differences calculations:

$$f_{i,\alpha} \sim -\left(\frac{A_{\text{perturb},\alpha} - A_0}{\Delta r_{i,\alpha}} \right)_{NVT}$$

where $\Delta r_{i,\alpha}$ denotes the perturbation of the ith atom along direction $\alpha \in (x, y, z)$, and $A_{\text{perturb},\alpha}$ is the free energy of the perturbed configuration. Similarly, the consistency between the energy and the stress tensor has been validated with finite differences as follows:

$$\sigma_{\alpha\beta} = \frac{1}{V} \left(\frac{\Delta A}{\Delta \varepsilon_{\alpha\beta}} \right) \Bigg|_{N,T,\varepsilon_{\{\alpha\beta\}}}$$

where ΔA denotes the difference of the free energy between the reference and the perturbed configuration which has been subjected to strain $\Delta\varepsilon_{\alpha\beta}$.

S2. Simulation protocol

The simulations were conducted with the EMSiPoN code[S3] for mesoscopic simulations. The equations of motion were integrated with the van Gunsteren and Berendsen scheme:[S4]

$$r_{i,\alpha}(t_n + \Delta t) = r_{i,\alpha}(t_n) + \frac{1}{\zeta_i} \left[F_{i,\alpha}(t_n) \Delta t + \frac{1}{2} \dot{F}_{i,\alpha}(t_n) (\Delta t)^2 \right] + R_{i,\alpha}(\Delta t) \quad \text{S19}$$

and the pressure was maintained with the Berendsen barostat.[S5] Since there are no velocities in Brownian Dynamics (high friction limit), the ideal gas contribution to pressure ($P_{\text{ig}} = n k_{\text{B}} T$) should be added to the total pressure explicitly in order to reproduce the correct density.

The following parameters were fixed to the corresponding values: N = 2000, T = 450 K, $N_A m_m$ = 14.02658 g/mol, $\zeta_m = 4.15\times10^{-13}$ kg/s and ρ_r = 766.947 kg/m^3. The parameters κ_r, N_c, N_m, P and Δt were varied throughout the simulations.

For each parameter combination, a simulation box was generated with N = 2000 particles at random positions. The initial volume of the box corresponds to the reference density:

$$V_{\text{sim,r}} = Nm / \rho_{\text{r}} = NV_{\text{r}} \qquad \text{S20}$$

with $m = N_m m_m$ being the particle mass. The cut off of the weighting kernel was set to:

$$r_{\text{c}} = \sqrt[3]{\frac{3}{4\pi} V_{\text{r}} N_{\text{c}}} \qquad \text{S21}$$

Note that the edge length of the simulation box is at least four times larger than the cutoff for the cases considered here:

$$\frac{\sqrt[3]{V_{\text{sim,r}}}}{r_{\text{c}}} = \frac{\sqrt[3]{NV_{\text{r}}}}{r_{\text{c}}} = \sqrt[3]{\frac{4\pi}{3}\frac{N}{N_{\text{c}}}}$$

Thus, finite-size effects are minimal.

In order to achieve a fair sampling regardless of the choice of Δt, we specify a characteristic sampling interval:

$$s_{\text{r}} = \left\lceil t_{\text{r/min}} / \Delta t \right\rceil \qquad \text{S22}$$

with $t_{\text{r/min}}$ = 0.25 ns being a constant time interval. The characteristic equilibration and sampling intervals were set to $s_{eq} = 1600\times s_r$ and $s_{sample} = 16000\times s_r$ steps, respectively. During the sampling phase of the simulation, the thermodynamic properties and trajectories are exported every $s_{thermo} = s_r$, and $s_{traj} = 160\times s_r$ steps, respectively. In doing so, the duration of the sampling phase is maintained to at least 4 μs for short time steps, and the export time intervals remain the same as well. Hence, the simulation outputs are rendered Δt-independent at short time steps and in this manner allow for conducting meaningful comparisons.

Initially, the free energy of the system was minimized with the implementation of the Conjugate Gradients algorithm[S6,S7] in EMSiPoN.[S8,S9] The sample was then equilibrated with Brownian Dynamics

for 10000 steps in the canonical ensemble (*NVT*) and then it was subjected to the isothermal-isobaric (*NPT*) ensemble until it achieved a thorough equilibration of density; hereafter the latter will be referred to as convergence phase. In order to automate the convergence phase and eliminate possible human bias we have implemented a convergence protocol which tracks the drift of the running average of the density during the course of the convergence phase. The protocol has as follows:

1) The sample is equilibrated for a waiting interval $s_{\text{delay}} = s_{\text{r}}$.
2) Subsequently, the running average of the density drift is estimated from the end of the waiting interval up to a correlation interval, s_{cor}:

$$\left\langle \Delta\rho_{\text{sim}}(s) \right\rangle = \sum_{s=s_{\text{wait}},s_{\text{wait}}+s_{\text{cor}}} \frac{\rho_{\text{sim}}(s) - \rho_{\text{sim}}(s-1)}{s_{\text{cor}}} = \frac{\rho_{\text{sim}}(s_{\text{wait}} + s_{\text{cor}}) - \rho_{\text{sim}}(s_{\text{wait}})}{s_{\text{cor}}} \qquad \text{S23}$$

3) After this correlation interval, the drift of the running averaged density is updated as follows:

$$\left\langle \Delta\rho_{\text{sim}}(s) \right\rangle = \lambda_{\text{cor}} \left[\rho_{\text{sim}}(s) - \rho_{\text{sim}}(s-1) \right] + (1 - \lambda_{\text{cor}}) \left\langle \Delta\rho_{\text{sim}}(s-1) \right\rangle \qquad \text{S24}$$

   where $\lambda_{\text{cor}} = 1/s_{\text{cor}}$.

4) Each time $\left\langle \Delta\rho_{\text{sim}}(s) \right\rangle$ changes sign, the event is recorded (a variable n_{swap} is incremented).
5) In case n_{swap} exceeds a prescribed maximum number ($n_{\text{swap\_max}}$), the process sends a convergence signal. If not, the convergence process is restarted from step 1.

In our case, the correlation steps were set to $s_{\text{cor}} = 800$, and the maximum sign swaps to $n_{\text{swap\_max}} = 10$. As a safety measure, after the convergence phase, the sample was equilibrated for an additional s_{equil} steps. Finally, a sampling period commenced for s_{sample} steps, during which the thermodynamic properties and the trajectories were exported every s_{r} and s_{traj} steps, respectively.

For demonstration purposes, Figure S1a illustrates the evolution of ρ_{sim} and $\left\langle \Delta\rho_{\text{sim}} \right\rangle$ during the convergence phase. Figure S1b and c depict the evolution of ρ_{sim} during the secondary equilibration and the sampling phase with respect to a semi-log and a normal *x*-axis (steps), respectively.

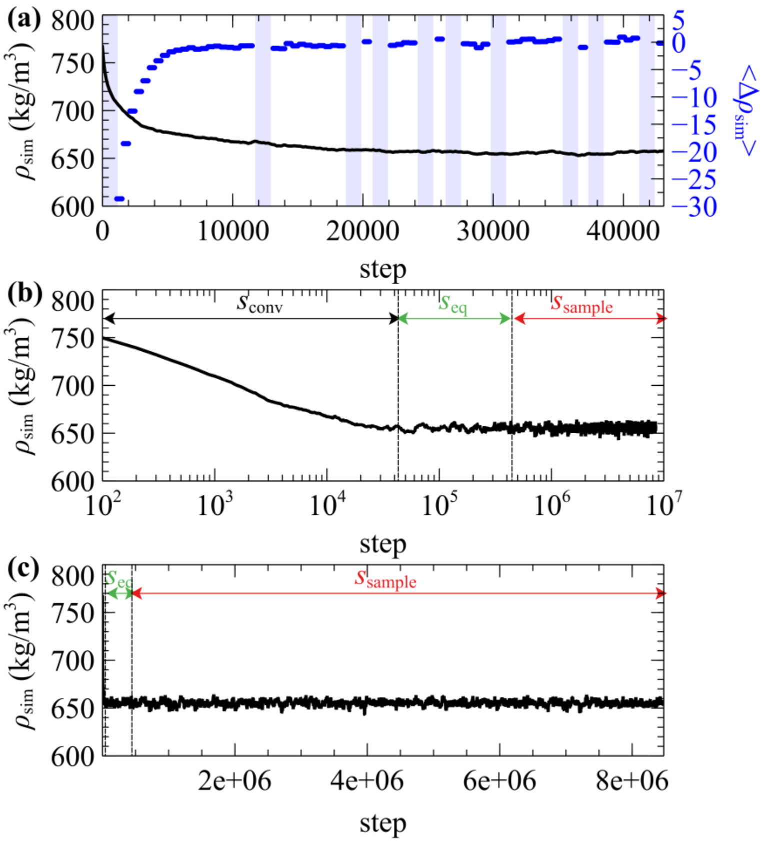

Figure S1. (a) Evolution of ρ_{sim} and $\langle \Delta\rho_{sim}(s) \rangle$ during the convergence phase. The ten shaded regions illustrate the waiting and density-drift-estimation intervals (s_{wait}+s_{cor}). **(b, c)** Evolution of ρ_{sim} during the secondary equilibration (s_{eq} = 401600 steps) and sampling (s_{sample} = 8032000 steps) phases. The parameters of this simulation are the following: Δt = 0.5 ps, N_m = N_c = 32, κ_r = 0.48881 GPa^{-1}, P = 1 atm.

S3. Validating the internal consistency of the reduced description

Table S1. Reduced pressure $\tilde{P}$ and total free energy ($\tilde{A} = \tilde{A}_{\mathrm{ex}} + \tilde{A}_{\mathrm{ig}}$) per-particle, at the beginning (subscript *init*) and averaged over the course of a simulation (subscript *ave*) of an FCC lattice with N = 2048 particles at density $n_{\mathrm{r}}=\rho_{\mathrm{r}}/(N_{\mathrm{m}}m_{\mathrm{m}})$ in the *NVT* ensemble for 10 steps with $\Delta t = \Delta t_{\mathrm{crit}}$ / 100. The parameter combinations in the first 7 columns yield the same reduced parameters $\tilde{\kappa}_{\mathrm{r}} = \kappa_{\mathrm{r}}\rho_{\mathrm{r}}k_{\mathrm{B}}T / N_{\mathrm{m}}m_{\mathrm{m}}$ = 1, $\tilde{r}_{\mathrm{c}}$ = 3.126 (or N_{c} = 128), and $\tilde{\zeta}/\Delta\tilde{t}$ = 0.0257; hence, the resulting pressures and free energies are the same. The first row depicts the reference values of these calculations. The red/black/green colors denote values which are 0.1, 1 and 10 times the reference values.

κ_{r} [GPa^{-1}]	ρ_{r} [kg/m^3]	T [K]	N_{m}	M_{m} [g/mol]	ζ_{m} [10^{-12} kg/s]	Δt [ps]	$\tilde{P}_{\mathrm{init}}$	$\tilde{P}_{\mathrm{ave}}$	$\tilde{A}_{\mathrm{init}}/N$	$\tilde{A}_{\mathrm{ave}}/N$
4.888	766.947	450	1	14.027	0.415	0.167	1.001	1.018	-1.000	-0.996
48.881	76.695	450	1	14.027	0.415	0.776	1.001	1.018	-1.000	-0.996
48.881	766.947	45	1	14.027	0.415	1.671	1.001	1.018	-1.000	-0.996
48.881	766.947	450	10	14.027	0.415	7.756	1.001	1.018	-1.000	-0.996
48.881	766.947	450	1	140.266	0.415	0.776	1.001	1.018	-1.000	-0.996
0.489	7669.470	450	1	14.027	0.415	0.036	1.001	1.018	-1.000	-0.996
4.888	7669.470	45	1	14.027	0.415	0.360	1.001	1.018	-1.000	-0.996
4.888	7669.470	450	10	14.027	0.415	1.671	1.001	1.018	-1.000	-0.996
4.888	7669.470	450	1	140.266	0.415	0.167	1.001	1.018	-1.000	-0.996
0.489	766.947	4500	1	14.027	0.415	0.017	1.001	1.018	-1.000	-0.996
4.888	76.695	4500	1	14.027	0.415	0.078	1.001	1.018	-1.000	-0.996
4.888	766.947	4500	10	14.027	0.415	0.776	1.001	1.018	-1.000	-0.996
4.888	766.947	4500	1	140.266	0.415	0.078	1.001	1.018	-1.000	-0.996
48.881	766.947	450	10	14.027	0.415	7.756	1.001	1.018	-1.000	-0.996
4.888	7669.470	450	10	14.027	0.415	1.671	1.001	1.018	-1.000	-0.996
4.888	766.947	4500	10	14.027	0.415	0.776	1.001	1.018	-1.000	-0.996
4.888	766.947	450	10	1.403	0.415	1.671	1.001	1.018	-1.000	-0.996
48.881	766.947	450	1	140.266	0.415	0.776	1.001	1.018	-1.000	-0.996
4.888	7669.470	450	1	140.266	0.415	0.167	1.001	1.018	-1.000	-0.996
4.888	766.947	4500	1	140.266	0.415	0.078	1.001	1.018	-1.000	-0.996
4.888	766.947	450	0.1	140.266	0.415	0.017	1.001	1.018	-1.000	-0.996
4.888	766.947	450	1	14.027	4.15	1.671	1.001	1.018	-1.000	-0.996
4.888	766.947	450	1	14.027	0.0415	0.017	1.001	1.018	-1.000	-0.996

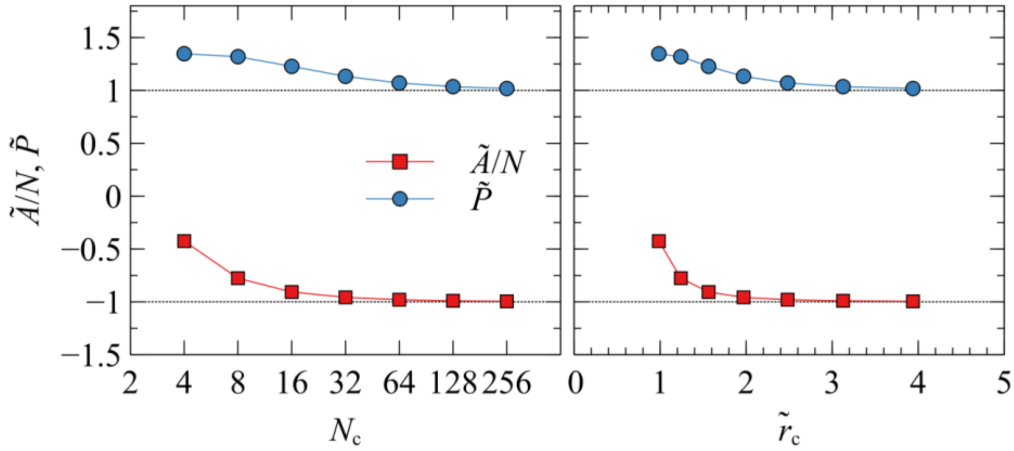

Figure S2. Reduced pressure ($\tilde{P}$, O) and total free energy ($\tilde{A} = \tilde{A}_{ex} + \tilde{A}_{ig}$, □) per-particle averaged over the course of a simulation of an FCC lattice with N = 2048 particles at density $\tilde{n}_r = 1$ in the NVT ensemble for 1000000 steps with Δt = Δt_{crit}/100, $\tilde{\kappa}_r = 1$, $\tilde{\zeta}/\Delta\tilde{t}$ = 0.0257 and varying (left) N_c or (right) $\tilde{r}_c$. Note that ideally, for $\tilde{n}_r = 1$, $\tilde{P}_{ex} = 0$ and thus $\tilde{P} = \tilde{P}_{ig} = \tilde{n}_r = 1$ (eq 20) and $\tilde{A}/N = \tilde{A}_{ig}/N = -1$.

REFERENCES

(S1) Wang, D. B.; Hsiao, F. Bin; Chuang, C. H.; Lee, Y. C. Algorithm Optimization in Molecular Dynamics Simulation. *Comput. Phys. Commun.* **2007**, *177* (7), 551–559. https://doi.org/10.1016/j.cpc.2007.05.009.

(S2) Welling, U.; Germano, G. Efficiency of Linked Cell Algorithms. *Comput. Phys. Commun.* **2011**, *182* (3), 611–615. https://doi.org/10.1016/j.cpc.2010.11.002.

(S3) Megariotis, G.; Vogiatzis, G. G.; Sgouros, A. P.; Theodorou, D. N. Slip Spring-Based Mesoscopic Simulations of Polymer Networks: Methodology and the Corresponding Computational Code. *Polymers (Basel).* **2018**, *10*, 1156. https://doi.org/10.3390/polym10101156.

(S4) van Gunsteren, W. F.; Berendsen, H. J. C. Algorithms for Brownian Dynamics. *Mol. Phys.* **1982**, *45* (3), 637–647. https://doi.org/10.1080/00268978200100491.

(S5) Berendsen, H. J. C.; Postma, J. P. M.; Van Gunsteren, W. F.; Dinola, A.; Haak, J. R. Molecular Dynamics with Coupling to an External Bath. *J. Chem. Phys.* **1984**, *81* (8), 3684–3690. https://doi.org/10.1063/1.448118.

(S6) Fletcher, R.; Reeves, C. M. Function Minimization by Conjugate Gradients. *Comput. J.* **1964**, *7* (2), 149–154. https://doi.org/10.1093/comjnl/7.2.149.

(S7) Polak, E.; Ribière, G. Note sur la convergence de méthodes de directions conjuguées. *ESAIM Math. Model. Numer. Anal. - Modélisation Mathématique Anal. Numérique* **1969**, *3* (R1), 35–43. https://doi.org/http://eudml.org/doc/193115.

(S8) Sgouros, A. P.; Theodorou, D. N. Addressing the Folding of Intermolecular Springs in Particle

Simulations : Fixed Image Convention. *Computation* **2023**, *11* (6), 106. https://doi.org/https://doi.org/10.3390/computation11060106.

(S9) Sgouros, P. A.; Theodorou, D. N. *FixImag*. https://github.com/ArisSgouros/FixImag.git.
